# Trimetazidine ameliorates hindlimb ischaemic damage in type 2 diabetic mice

**DOI:** 10.1080/07853890.2021.1925147

**Published:** 2021-07-14

**Authors:** Yan Yang, Qinqin Xu, Tao Li, Shiying Shao

**Affiliations:** a Division of Endocrinology, Tongji Hospital, Huazhong University of Science & Technology, Wuhan, PR China; b Branch of national clinical research center for metabolic diseases, Hubei, PR China; c Division of Ophthalmology, Tongji Hospital, Huazhong University of Science & Technology, Wuhan, PR China

**Keywords:** Diabetes, peripheral artery disease, trimetazidine, angiogenesis

## Abstract

**Background:**

Ischaemia caused by lower extremity artery stenosis is the main cause of peripheral artery disease (PAD) in patients with diabetes. Trimetazidine (TMZ) has traditionally been used as an anti-ischaemic drug for coronary artery disease. The effect of TMZ on PAD in a diabetic animal model and the underlying molecular mechanisms remain unclear.

**Methods:**

The db/db mice were challenged with femoral artery ligation (FAL), followed by TMZ treatment for 2 weeks. Scores on hindlimb ischaemia and function were evaluated. Histological and capillary density analyses of gastrocnemius were performed. The expression of vascular endothelial growth factor (VEGF) and myogenic regulators was also confirmed by Western blotting. We also detected serum intercellular adhesion molecule 1 (ICAM-1) level through ELISA.

**Results:**

Diabetic mice exhibited limb ulceration and motor dysfunction after FAL while TMZ-treated db/db mice exhibited milder ischaemic impairment. Furthermore, decreased capillary density in the gastrocnemius muscles of ischaemic hindlimb and reduced expressions of VEGF, myogenic markers, and serum ICAM-1 could be partially reversed by TMZ treatment.

**Conclusion:**

TMZ may alleviate hindlimb ischaemic damage in db/db mice, at least partly, through enhancing angiogenesis and promoting myogenesis in ischaemia region.

**Key messages**
- TMZ intervention could alleviate hindlimb ischaemic damage in db/db mice.
- TMZ intervention could enhance angiogenesis and stimulate myogenesis in ischaemia region.

## Introduction

Peripheral arterial disease (PAD) is a widespread disease and the obstruction to blood flow in the lower extremity is a major concern [1]. One of the most important risk factors of lower extremity PAD is diabetes. In population studies of patients with PAD, about 20% have diabetes; parallelly, approximately 20% to 26% of diabetic patients are complicated with PAD [2,3]. Diabetes has been associated with a 2- to 4-fold elevation in the prevalence of PAD [4].

Moreover, the presence of diabetes not only increases the incidence of PAD but also accelerates disease progression and worsens disease severity [4]. The main clinical manifestations of PAD include intermittent claudication, rest pain, and even ischaemic ulcers due to tissue hypoperfusion [1]. It is found that subjects with diabetes and PAD have more severe outcomes including amputations and mortality [5,6]. The estimated percentage of diabetes ranges from 27% to 76% among the PAD patients with chronic limb-threatening ischaemia (CLTI) [7].

Open surgery and endovascular technology are currently considered as the first-line choices for revascularization of PAD [8]. However, the clinical outcomes are highly dependent on the anatomical pattern of the disease. There are limited medical therapies that have direct benefits to reduce limb-related adverse events, improve quality of life, and enhance performance status in advanced PAD [9,10].

Trimetazidine (TMZ), a piperazine derivative agent, was synthesized in 1969 in Servier Laboratories (France). By selectively inhibiting the activity of enzyme 3-ketoacyl coenzyme A thiolase (3KAT) in the β-oxidation pathway, TMZ enhances glucose metabolism of cardiomyocytes and maintains energy production with less oxygen consumption [11]. Due to its significant anti-ischaemic properties [12], TMZ has become a standard treatment for ischaemic cardiomyopathy (ICM) [13] as monotherapy or adjunct therapy since 1978 [14,15]. Nowadays, TMZ is prescribed as a long-term treatment of angina pectoris in more than 90 countries around the world [16].

Accordingly, most of previous clinical and pre-clinical studies explore the protective effects of TMZ on cardiomyocytes. Liu et al. reported that TMZ treatment significantly activated AMP-activated protein kinase (AMPK) signalling and modulated substrate metabolism by shifting fatty acid oxidation to glucose oxidation, leading to reduction of oxidative stress in the ischaemia/reperfusion hearts [17]. In addition, early administration of TMZ could attenuate diabetic cardiomyopathy by reducing cardiomyocyte apoptosis and restoring cardiac autophagy [18].

Recent investigations identified that, beyond the treatment of cardiomyopathy, TMZ may play an important role in diverse clinical situations including PAD, contrast-induced nephropathy, and reperfusion injury [19]. Several studies have evaluated the role of TMZ in the treatment of PAD. One such study published in 2003 proved the ability of TMZ to extend the distance of intermittent claudication in patients with atherosclerosis obliterans [20]. Similarly, Vitale et al. reported a significant improvement of maximal walking distance in patients with PAD after TMZ intervention [21], indicating a promising role of TMZ in PAD. However, the effect of TMZ on ischaemic vascular disease in diabetes is not clear. In this study, we sought to investigate the potential therapeutic impact of TMZ on ischaemic damage in db/db mice and to elucidate the underlying mechanisms.

## Materials and methods

### Femoral artery ligation and TMZ intervention

Seven-week-old male db/db (C57BLKS/J-lepr^db^/lepr^db^) mice (Model Animal Research Centre of Nanjing University, Nanjing, China) were fed with a standard chow diet ad libitum and had free access to drinking water. Fasting blood glucose levels were measured every 3 d. Mice with blood glucose above 16.7 mmol/L were underwent left femoral artery ligation (FAL) as previously reported [22]. Briefly, after anesthesia, the femoral artery was isolated and ligated with 5-0 silk sutures at proximal and distal places (keeping the same distance in all mice). After FAL, mice were given TMZ (10 mg/kg, Sigma-Aldrich, St. Louis, MO, TMZ group, *n* = 10) or an equal volume of saline solution (saline group, *n* = 10) by intragastric administration every day for 2 weeks. Sham surgery was performed by passing the suture underneath the femoral artery without ligation (Control group, *n* = 10). All animal care and experimental procedures were approved by the Institutional Animal Care and Use of Committee of Tongji Medical College of Huazhong University of Science & Technology.

### Assessment of limb ischaemia

To determine the progress and severity of the lesion in ischaemic hind limb, a five-point scoring system, described by Stabile and Wang, was utilized [23,24]. Any evidence of post-FAL amputation was scored as 5; severe discolouration or tissue necrosis was scored as 4; diffuse ulceration was scored as 3, and mild discolouration (pale appearance) or local ulceration was scored as 2. A completely normal appearance of the limb compared to the non-FAL limb was rated 1. The ischaemic scores were evaluated 14 d after ligation.

### Assessment of limb function

A semi-quantitative estimation of limb function was performed serially using the following classification. Dragging of the foot (foot necrosis) was scored as 3; no dragging but no plantar flexion (foot damage) was scored as 2; plantar flexion but no toe flexion (toe damage) was scored as 1; flexing the toes to resist gentle traction on the tail (no damage) was scored as 0 [23].

### *In-vivo* capillary density and muscle regeneration measurement

Mice were sacrificed 14 d after FAL to collect ischaemic left gastrocnemius muscle. 7-μm thick cryosections were first blocked with 5% normal goat serum and then incubated with primary antibodies CD31 (a marker for endothelial cell, Santa Cruz, CA) and α-smooth muscle actin (α-SMA) (Abcam, UK, Cambridge) at 4 °C overnight, followed by the incubation with secondary antibodies Alexa-488 conjugated anti-rabbit IgG and Alexa-594 conjugated anti-mouse (1:500; Jackson ImmunoResearch Laboratories, PA, USA) for 1 h at room temperature. Subsequently, sections were mounted using Vectashield with DAPI (4'6-diamino-2-fenilindol dihidrocloreto) for counter-staining of nuclei before observation. Pictures from each section were taken under 400× magnification, using Olympus BX51 high-magnification microscope. Capillaries were identified by positive staining for CD31 and those displaying a second cellular layer stained with SMA, surrounding the inner one, were counted as arterioles. According to the procedures previously published [25,26], ten different fields from each tissue section were selected randomly. Capillaries labelled with CD31 were counted. Capillary density was expressed as the number of capillaries per square millimetre. The proportion of fibres with central nucleus (regenerated fibres) in the injured area was calculated [27].

### Western blotting analysis

A total of 30 μg protein samples from mice gastrocnemius lysates were separated on a 10% sodium dodecyl sulphate polyacrylamide gel electrophoresis (SDS-PAGE) and electrophoretically transferred onto polyvinylidene difluoride (PVDF) membranes. The membranes were incubated with 5% non-fat dry milk in Tris-buffered saline Tween-20 (TBST) for 2 h and then incubated overnight at 4 °C with primary antibodies including vascular endothelial growth factor (VEGF)-A (1:1000, Servicebio, Wuhan, Hu Bei, China), myogenin (1:1000, Abcam, Cambridge, MA, USA), and Myf5 (1:1000, Abcam). The blots were developed with an enhanced chemiluminescence reagent kit. The intensities of individual bands were analyzed by densitometry using IMAGEJ (National Institutes of Health Software, Bethesda, MD, USA). All groups were normalized to their respective controls, and bar graphs represent quantification of at least 3 independent experiments.

### ELISA analysis

Serum intercellular adhesion molecule 1 (ICAM-1) level was measured using ELISA kits (Bio-Rad Laboratories, USA) according to the manufacturer's instruction.

### Statistical analysis

Data were presented as mean ± SEM. Data analysis was performed with GraphPad Prism 6.0 Software. Comparisons between groups were performed by one-way ANOVA followed by Newman–Kuels multiple comparison test. Ischaemic and function scores were analyzed using two-way ANOVA, followed by the post hoc test with the Bonferroni correction for multiple comparisons. The results were considered significant at *p* < .05.

## Results

### TMZ protects db/db mice against hindlimb ischaemia

**Figure 1.**
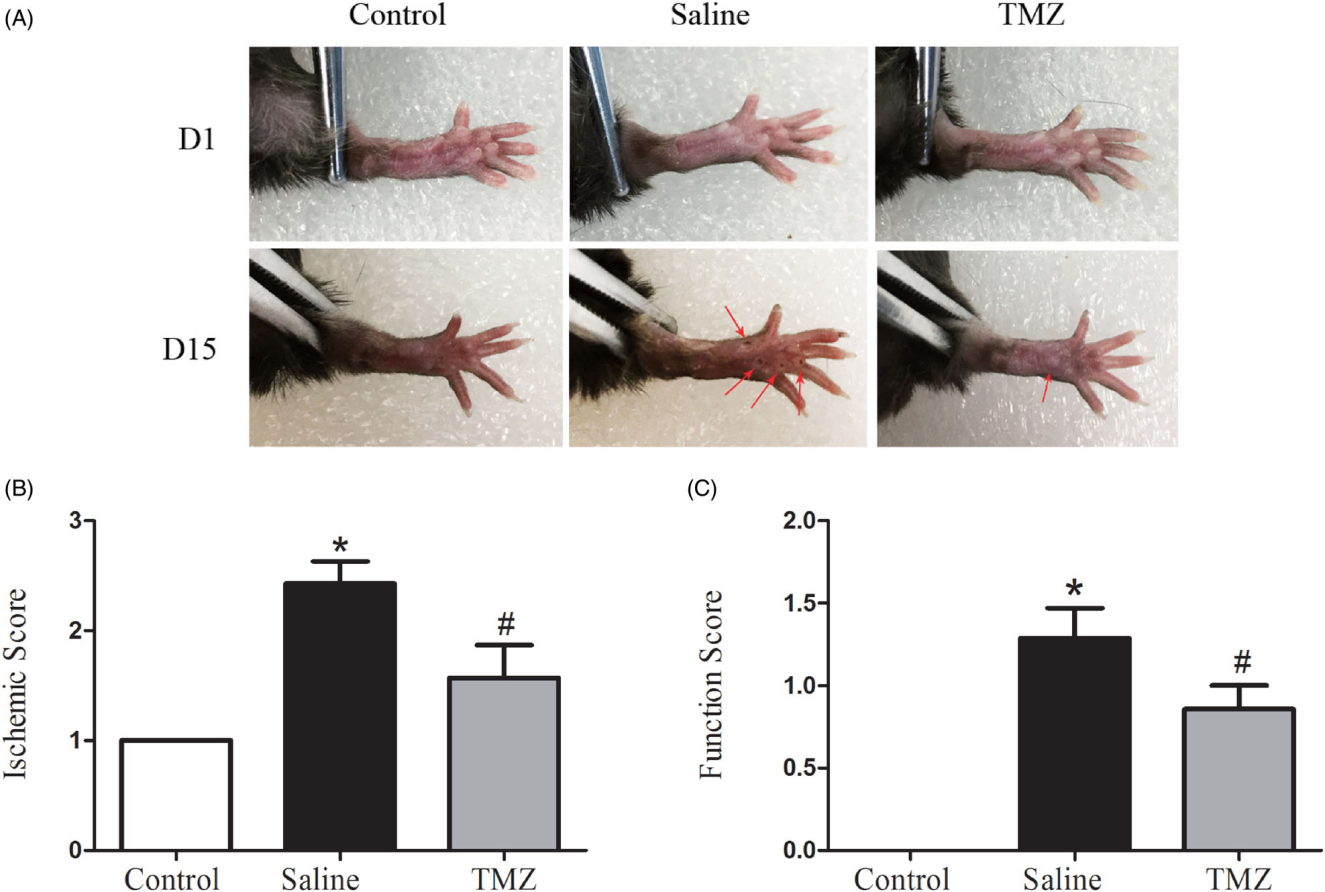
TMZ attenuates ischaemic injury in hindlimb of diabetic mice. (A) Representative photographs of hindlimbs from mice with sham surgery (control group, *n* = 10), untreated mice after FAL (saline group, *n* = 10), and TMZ-treated mice after FAL (TMZ group, *n* = 10) were shown; (B) The severity of the lesion in ischaemic hindlimb was estimated according to a five-point scoring system; (C) The foot function score of the ischaemic hindlimb was estimated. Results are expressed as mean ± SEM. *p < .05 compared to the control group, ^#^*p* < .05 compared to the saline group.

Two weeks after FAL, the effect of TMZ on foot ulceration and claudication was investigated. The db/db mice with hindlimb ischemia exhibited severe ulceration and increased ischaemic score (2.43 ± 0.20, *p* < .01) compared to those in the sham surgery group (Figure 1(A,B)). TMZ intervention significantly improved the ischaemic damage (ischaemic score: 1.57 ± 0.30, *p* < .01). In order to assess the motor functional benefit of TMZ, the function scores were assessed as well. Diabetic mice exhibited impaired motor function (Figure 1(C)) although none of the mice exhibited foot-dragging. TMZ-treated db/db mice presented slighter motor impairment when compared with the saline group (TMZ group 0.86 ± 0.14 vs. saline group 1.29 ± 0.18, *p* < .05). Together, these findings clearly demonstrate that TMZ therapy is effective in restoring ischaemia-induced hindlimb damage in diabetic mice.

### TMZ enhances angiogenesis in ischaemic hindlimb

**Figure 2.**
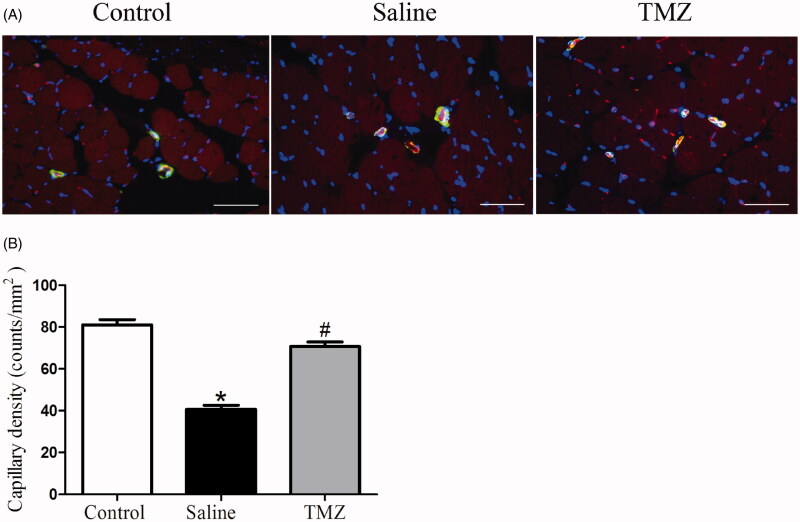
TMZ therapy promotes angiogenesis in the ischaemic hindlimb of diabetic mice. (A) Sections of ischaemic muscles recovered at day 14 post-surgery were stained for CD31 (endothelial stain; red), α-SMA (smooth muscle strain; green), and DAPI (nuclear stain; blue). Representative immunofluorescence of muscle sections were shown (*n* = 3 sections per mouse, 10 mice per group). Scale bar: 50 μm; (B) Quantitative analysis of capillary density in gastrocnemius muscle was performed. Results are expressed as mean ± SEM. *p < .05 compared to the control group, ^#^*p* < .05 compared to the saline group.

Angiogenesis was examined by immunocytochemistry at day 14 post-ligation to understand the effect of TMZ on ischemic injury. The tissue sections were stained with CD31 and α-SMA to visualize capillary and arteriole density. Indeed, significantly decreased numbers of capillary were observed in the gastrocnemius muscle of ischaemic hindlimbs while TMZ intervention prevented rarefaction of capillary vessels (Figure 2(A)). As shown in Figure 2(B), TMZ markedly restored the capillary density in the ischaemic region of the diabetic mice to a nearly normal level (TMZ group 70.80 ± 2.18 vs. saline group 40.60 ± 1.91, *p* < .01). However, TMZ had no effect on arteriolar density (data not shown). Taken together, these results demonstrate that the capillary regeneration in diabetic ischaemic hindlimb is augmented by TMZ treatment.

### TMZ normalizes the expressions of VEGF-A and ICAM-1in ischaemic hindlimb

**Figure 3.**
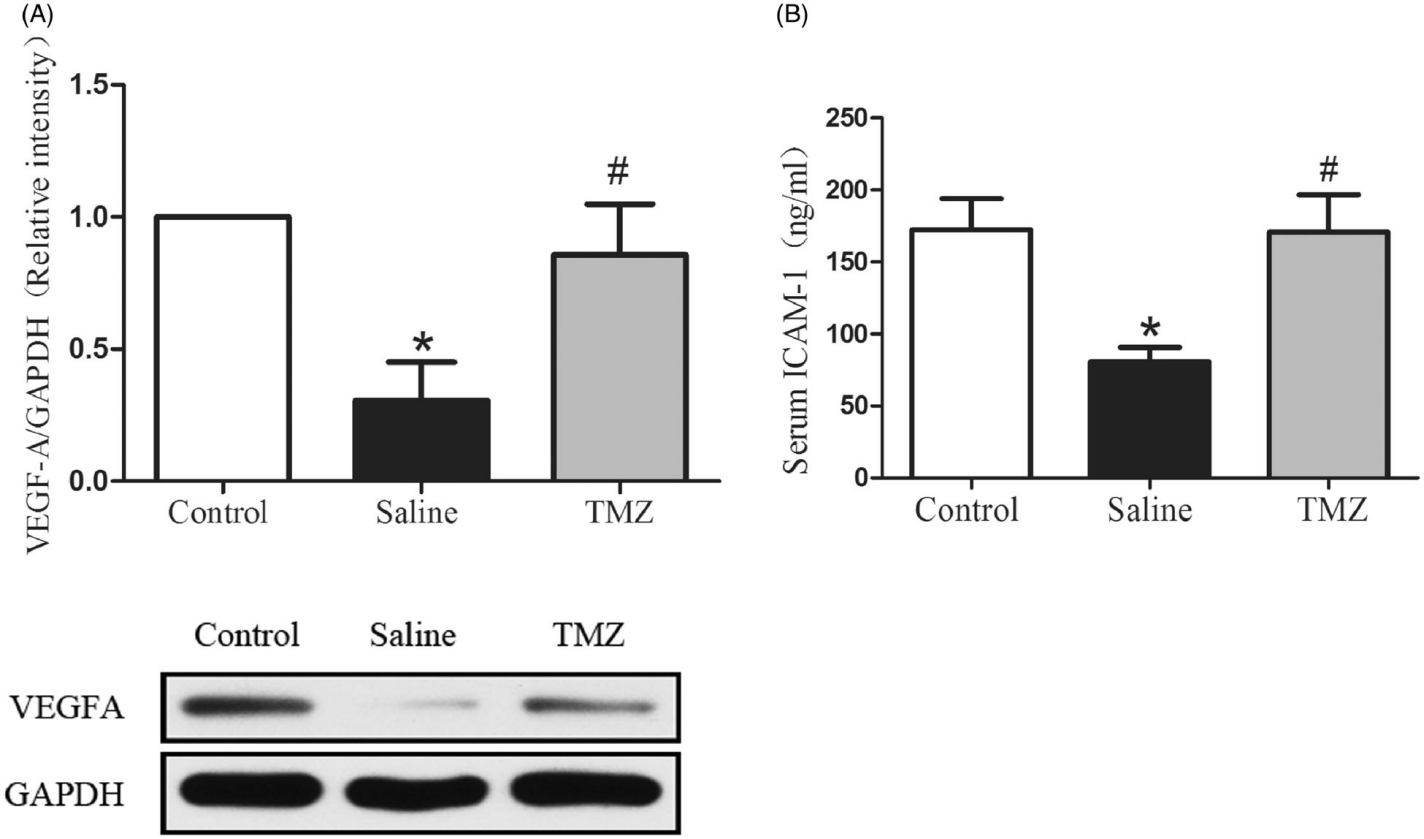
TMZ stimulates VEGF-A expression in the ischaemic muscles and elevates serum ICAM-1 level. (A) Gastrocnemius extracts were assayed for VEGF-A protein levels by immunoblot and quantitation; (B) Serum levels of ICAM-1 were measured by ELISA. Data are shown as means ± SE. *p < .05 compared to the control group, ^#^*p* < .05 compared to the saline group.

VEGF-A activation plays a critical role in the induction of the angiogenesis process [28]. To further investigate the mechanism by which TMZ augments angiogenesis in diabetic mice with ischaemic damage, expression of VEGF-A was evaluated two weeks after the ligation. As illustrated in Figure 3(A), the expression of VEGF-A in diabetic mice decreased by 69% compared with the control group. Of note, the administration of TMZ up-regulated VEGF-A expression by 1.77-fold (TMZ group 0.86 ± 0.19 vs. saline group 0.31 ± 0.14, *p* < .05). ICAM-1 is an adhesion molecule that could also mediate angiogenesis [29]. Thus, we further investigated the serum level ofICAM-1. As shown in Figure 3(B), serum ICAM-1 level was significantly decreased by 47% in the hindlimb ischaemia model compared with that in the control group and this decrease was reversed in TMZ-treated diabetic mice.

### TMZ enhances muscle regeneration in ischaemic hindlimb

**Figure 4.**
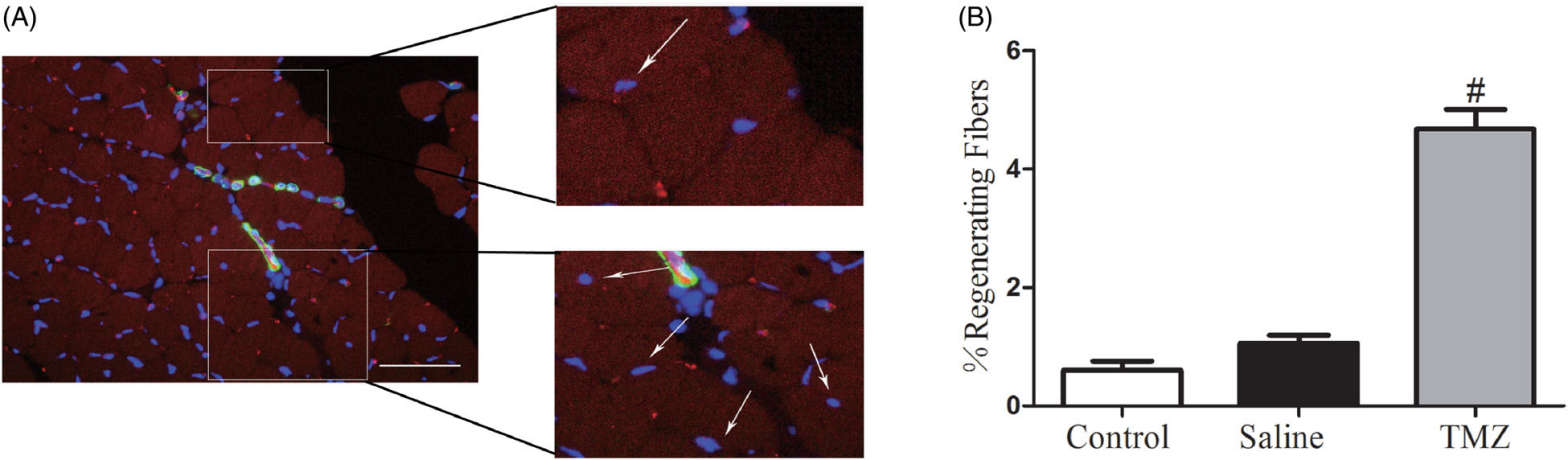
TMZ therapy promotes myofiber regeneration in the ischaemic hindlimb of diabetic mice. (A) Immunofluorescence of muscle sections from TMZ-treated mice with CD31 (endothelial stain; red), α-SMA (smooth muscle stain; green) and DAPI (nuclear stain; blue). Scale bar: 50 μm. White arrows indicate regenerating myofibers, characterized by central nucleus location; (B) Quantification of the percentage of regenerating fibres, characterized by the presence of a centrally located nucleus. Results are expressed as mean ± SEM. ^#^*p* < .05 compared to the saline group.

Muscle regeneration was examined at day 14 post-ligation and fibres with central nucleus were considered as regenerated muscle [27]. As shown in Figure 4, TMZ significantly increased the number of regenerating myofibers in the ischaemic hindlimb of diabetic mice (TMZ group 4.68 ± 0.33% vs. saline group 1.06 ± 0.14%, *p* < .05), indicating its potential capability to stimulate myogenesis.

### TMZ normalizes the expressions of myogenic regulators in ischaemic hindlimb

**Figure 5.**
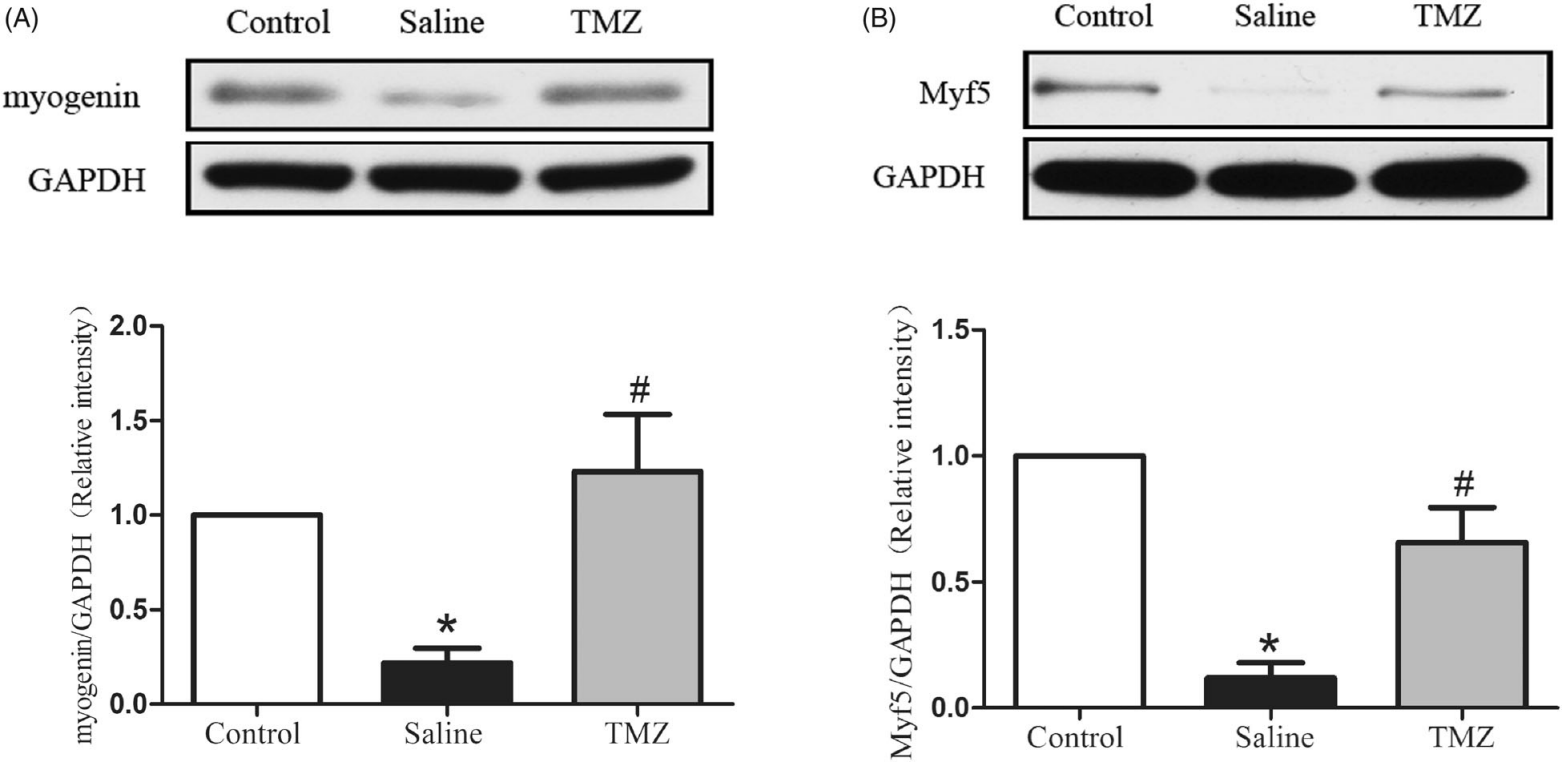
TMZ reverses the changes of myogenic markers in the ischaemic hindlimb of diabetic mice. Immunoblot and quantitation of (A) myogenin and (B) Myf5 were assayed. Data are shown as means ± SE. *p < .05 compared to the control group, ^#^*p* < .05 compared to the saline group.

We further detected the expression of myogenin and Myf5, two myogenic markers. The result indicated an obvious decrease of myogenin in PAD model (0.22 ± 0.08 vs. control group, *p* < .05) while administration of TMZ significantly increased myogenin expression in the ischaemic hindlimb of diabetic mice. We also detected the decreased expression of Myf5, which could be corrected by TMZ administration (TMZ group 0.66 ± 0.14 vs. saline group 0.12 ± 0.06, *p* < .01) (Figure 5(A,B)). Collectively, these results indicate that TMZ have the potential to enhance the regeneration of ischaemic muscles in diabetic mice through myogenic regulation pathway.

## Discussion

It is well known that TMZ is a conventional anti-angina drug. Beyond the traditional application in ICM, TMZ shows a promise in the treatment of PAD by extending the intermittent claudication distance [20,21]. In the present study, we demonstrated the curative effects of TMS on ischaemic hindlimbs in diabetic mice. TMZ improved neovascularization as manifested by the increased vascular density and VEGF-A expression. In addition, TMZ promoted the expression of myogenic markers in ischaemia region, indicating the potential benefit in myogenesis. To our knowledge, this is the first study demonstrating the important role and mechanism of TMZ in restoring hindlimb ischaemic injury in the diabetic animal model. These findings support the direct evidence of TMZ in diabetic PAD and contribute to enlarge its scope of application in the clinic.

Revascularization is a complicated process involving stimulation of endothelial cell signal pathway activation, proliferation, extracellular matrix remodelling, and vessel maturation [30,31]. Of note, impaired angiogenesis can be observed in diabetes which hinders wound healing and coronary collateral vessel development [32,33]. Hu et al. reported that intervention of TMZ with mesenchymal stem cells could significantly promote neovascularization and improve cardiac function of rats subjected to myocardial ischaemia/reperfusion (I/R) injury [34]. Our current data identified that TMZ treatment significantly increased vascular density in ischaemic tissue in diabetic mice, indicating the potential effect of TMZ on neovascularization in the ischaemic condition of diabetes.

It is well known that VEGF acts as an essential regulatory factor in the process of neovascularization by promoting collateral vessel formation, endothelial cell recruitment, proliferation and differentiation [28]. VEGF level decreased significantly in diabetic mice with a fracture [35]. In addition, the development and function of coronary collateral vessels were also reduced in patients with diabetes, which may be attributed to the altered chemotactic response of monocytes to VEGF (VEGF resistance) [36]. Thus, it is speculated that promoting revascularization by upregulating angiogenic VEGF may contribute to the restoration of blood perfusion into ischaemic tissues in subjects with diabetes and PAD. Our results also observed the decrease of VEGF-A in the ischaemic region and TMZ administration significantly reversed the reduced VEGF-A level, which provides experimental evidence for the clinical application of TMZ in the treatment of ischaemic PAD. Furthermore, VEGF-A is known to present at least four splice variants including VEGF121, VEGF165, VEGF189, and VEGF206 [37,38]. VEGF121b, VEGF165b and VEGF189b variants show anti-angiogenic effect whileVEGF121a and VEGF165a variants exert pro-angiogenic effect [39]. It would be of significance to investigate the effect of TMZ on pro-angiogenic and anti-angiogenic VEGF variants.

Interestingly, in the current study, we identified the serum level of ICAM-1 (sICAM-1) was significantly decreased after hindlimb ischaemic injury (Figure 3(B)). The ICAM-1 protein belongs to the immunoglobulin superfamily synthesized by endothelial cells, the predominant function of which is the recruitment and trafficking of leukocytes *via* interactions with leukocyte-expressed integrins [40]. Previous reports have shown that ICAM-1 is also implicated in the development of inflammatory vascular disease and plays an important role in angiogenesis [29,41]. It was found that ICAM-1−/− mice displayed no increase in capillary density in response to VEGF-A, indicating an essential role of ICAM-1 in modulating VEGF-induced angiogenesis [29]. Furthermore, we identified that TMZ treatment could elevate the level of sICAM-1 (Figure 3(B)). Thus, it is speculated that TMZ, to a certain extent, may promote VEGF induced angiogenesis via modulating sICAM-1generation. Different from our findings, a small randomized study including 18 patients with acute myocardial infarction assessed the effect of TMZ on plasma ICAM-1 levels, which identified that the plasma ICAM-1 level of the TMZ group was elevated for the first 24 h but decreased in the next two days [42]. The relevant researches are limited and studies with a longer observation period and the larger sample size are necessitated. In addition, the detailed mechanism of TMZ in regulating sICAM-1 is not clear, which will be explored in future work.

Of note, many studies identified that TMZ may exert its protective effect in ischaemic injury *via* regulating the expression of hypoxia-inducible factor-1α (HIF-1α), which could attenuate oxidative stress *via* expression of VEGF [34,43,44]. Park et al. reported that TMZ intervention contributed to the recovery of renal dysfunction after I/R injury, which may be mediated by the up-regulation of HIF-1α and its downstream target VEGF [45]. Further, investigation to identify whether the elevation of VEGF in ischaemic hindlimb is also regulated by HIF-1α or other potential molecules would be significant.

Impaired angiogenesis also affects skeletal muscle regeneration in the condition of diabetes [46]. Here, our data showed that TMZ treatment protected against ischaemia-induced motor dysfunction in diabetic mice after FAL, which may be partly attributed to the improvement of angiogenesis and blood flow. Furthermore, myogenin and Myf5 are members of the family of myogenic regulatory factors and play a key role in myogenic differentiation and muscle regeneration [47,48]. Our result also identified decreased expression of these two myogenic proteins (Figure 4). Gatta et al. confirmed that the metabolic modulator TMZ promoted myoblast differentiation and enhanced new myofiber formation in a mice model of cancer cachexia [49]. This process may be mediated by the phosphorylation of AMPK and up-regulation of the peroxisome proliferator-activated receptor-gamma coactivator 1-α (PGC1α), both of which could promote myoblast differentiation by enhancing the mitochondrial biogenesis [49]. Another study revealed the ability of TMZ to stimulate myogenesis *via* triggering myogenic gene expression including MyoD, myogenin, and desmin in aged mice [50]. Consistently, TMZ administration could markedly correct the decreased levels of myogenin and Myf5 in ischaemic hindlimb. We also detected the expression level of MyoD, which was decreased significantly as well. But TMZ administration did not reverse it (data not shown). These findings suggest that, besides the indirect effect on angiogenesis to ameliorate ischaemia-induced motor dysfunction, TMZ may also directly stimulate the regeneration of ischaemic muscles in db/db mice by increasing the levels of myogenin and Myf5.

In conclusion, this study demonstrates that TMZ therapy notably ameliorated foot injury in the ischaemic hindlimb in diabetic mice and improved angiogenesis via regulating the expression of ICAM-1 and VEGF-A. In addition, TMZ may also increase the myogenic regulators and thus stimulate myogenesis. These data highlight the potential utility of TMZ therapy as a supplementary non-invasive treatment for diabetic PAD.

## Data Availability

The data supporting the findings of this study are available within the article.
